# Young Children’s Sugar-Sweetened Beverage Consumption and 5-Year Change in BMI: Lessons Learned from the Timing of Consumption

**DOI:** 10.3390/nu12082486

**Published:** 2020-08-18

**Authors:** Petra C. Vinke, Karlien A. Blijleven, Milou H. H. S. Luitjens, Eva Corpeleijn

**Affiliations:** Department of Epidemiology, University Medical Center Groningen, University of Groningen, 9700 RB Groningen, The Netherlands; karlienblij@gmail.com (K.A.B.); milouluitjens@gmail.com (M.H.H.S.L.); e.corpeleijn@umcg.nl (E.C.)

**Keywords:** sugar-sweetened beverages, childhood overweight, eating pattern, eating occasion, parental involvement, prospective cohort

## Abstract

Sugar-sweetened beverages (SSBs) are an already known risk factor for weight gain in childhood. To identify windows of opportunity for public health interventions, insight into the consumption behavior of SSBs is needed. We investigated whether total SSB consumption was related to body mass index (BMI) change and overweight and compared whether the timing of consumption over the day differed between low and high consumers. In the Dutch GECKO Drenthe birth cohort, a cohort embedded within the Groningen Expert Center for Kids with Obesity (GECKO), height and weight were measured by trained nurses at age 5/6 years (y) and 10/11 y (*N* = 1257). BMI was standardized for age and sex (BMI-z). In the food pattern questionnaire completed by parents at age 5/6 y, beverages were assessed for seven time segments (breakfast, morning, lunch at school, lunch at home, afternoon, dinner, and evening). Linear and logistic regression analyses were adjusted for potential confounders (sex, baseline BMI-z, parental BMI, parental education level, maternal age at birth, maternal smoking during pregnancy). The median daily SSB consumption frequency ranged from 1.9 times per day (1.5–2.0, 25th–75th percentile) in the lowest quartile to 4.9 times per day (4.6–5.5) in the highest quartile. In the highest compared to the lowest quartile of SSB consumption frequency, the confounder-adjusted odds ratio for overweight incidence was 3.12 (95% CI, 1.60–6.07). The difference in consumption between quartile 1 and quartile 4 occurred mainly during main meals and in the evening, e.g., at breakfast (31% vs. 98%, *p* < 0.001), lunch at home (32% vs. 98%, *p* < 0.001), and dinner (17% vs. 72%, *p* < 0.001). These drinking occasions characterizing high SSB consumers mostly occurred in the home environment, where parental influence on dietary behaviors is profound. Therefore, these results exposed a window of opportunity, leading to the advice for parents to offer their children sugar-free drinks to quench thirst with main meals.

## 1. Introduction

Over the past decades, the global prevalence of overweight and obesity in children has increased dramatically [1]. Even in childhood, overweight increases the risk of hyperlipidemia, hypertension, and type 2 diabetes [2] and is a strong predictor for adult overweight and its associated health consequences [3]. Sugar-sweetened beverages (SSBs) are a well-known nutritional risk factor for excess weight gain in children [4], and have, therefore, frequently been targeted in intervention studies. However, educational, behavioral, and environmental intervention studies in children so far have only succeeded in moderate decreases in SSB intake [5,6], leaving substantial room for improvement. In order to optimize intervention strategies, insight into the consumption patterns of SSBs in children can help to identify targets for intervention that can be harmonized with established eating habits. 

A positive energy balance is a key factor in excess weight gain. Sugar-sweetened beverages not only contribute to a positive energy balance and subsequent weight gain because of their high energy density just like other sugar-rich food items but also due to their low satiating capacity. It was shown that daily intake of one SSB portion does not elicit full caloric compensation, while the daily intake of an equicaloric serving of candy does [7]. A suggested underlying mechanism is that liquid calories might elicit fewer satiety signals to the brain and the digestive tract, resulting in an impaired registration of the incoming calories [8]. As SSBs are already consumed at a young age [9,10], they became a frequently studied target for early life prevention of overweight.

Prospective cohort studies in young children between age 2 and 10 years [11,12,13] as well as a randomized controlled trial including children between age 4 and 11 years [14] have confirmed that SSB consumption significantly contributes to weight gain in early childhood. Interventions to limit SSB consumption in young children, for example implemented in healthcare, home, and preschool settings, have had modest effects [5,6,15]. Furthermore, the effect sizes of interventions with a duration of 30 weeks or longer were found to be lower than those for interventions with a duration below 30 weeks (mean difference −36 vs. −158 mL/day) [5]. This is an indication that interventions may not succeed in sustainable changes in SSB consumption behaviors. Better insight into the habitual timing of SSB consumption in young children, may help to identify targets for interventions that are more easily implemented within habitual eating patterns. This could help to improve the (long-term) efficacy of interventions and with this decrease childhood overweight. 

In this study, the timing of SSB consumption throughout the day was examined in children with varying levels of total SSB intake. First, we investigated the association between SSBs and change in body mass index (BMI) and overweight incidence, to subsequently investigate whether SSB intake at specific time segments, or in specific patterns over the day, distinguishes children with high SSB intake from those with low intake. These insights can help identify windows of opportunity for behavioral or environmental interventions in the home or school environment aiming to decrease SSB intake in children, by learning from the behaviors of children who already succeed in consuming SSBs in moderation. 

## 2. Materials and Methods

### 2.1. GECKO Drenthe Birth Cohort and Study Population

The GECKO Drenthe birth cohort, a cohort embedded within the Groningen Expert Center for Kids with Obesity (GECKO), was initiated to examine the determinants and development of childhood overweight in Dutch children. The study design and set up were previously described in detail elsewhere [16]. All pregnant women in the province of Drenthe, in the north of The Netherlands were invited to participate by obstetricians, midwives, and general practitioners in the third trimester of their pregnancy. The cohort includes 2997 children born in 2006 and 2007. Participating families are representative for the study area with regard to overweight and education levels of parents, but ethnic minorities were slightly underrepresented (9.1% in the area’s population vs. 6.0% in the cohort). For the current study, children with a birth weight below 2500 g and children for whom data on SSB consumption, potential confounders, or BMI at age 5/6 or 10/11 years was missing or incomplete were excluded from the analyses (Figure S1). Written informed consent was obtained from all parents. The GECKO Drenthe birth cohort was approved by the Medical Ethics Committee of the University Medical Center Groningen (2005/260) and follows the guidelines of the Declaration of Helsinki. The cohort is registered at www.birthcohorts.net, the research question with analysis plan for the current study was not specified within this registration. The manuscript is an accurate and transparent account of the study, and no important aspects of the study or any analyses conducted have been omitted.

### 2.2. Data Collection

#### 2.2.1. Anthropometrics

Birth weight rounded to the nearest 5 g was reported by midwives. Trained nurses at the Community Health Services (GGD) performed anthropometric measurements around age 5/6 and 10/11 years. Height and weight were measured to the nearest 0.1 cm or kg, respectively. 

#### 2.2.2. Parental Information

During pregnancy, questionnaires were issued to both parents to assess pre-pregnancy BMI (kg/m^2^), maternal smoking during pregnancy (yes/no), and parental age and education level at birth. Education level was categorized as low (no education, primary school, lower vocational, or lower general secondary education), middle (intermediate vocational training or higher secondary education), or high (higher vocational or university education).

#### 2.2.3. Food Pattern Questionnaire

To assess the children’s diets, parents filled in a Food Pattern Questionnaire (FPQ) at 5/6 years. The questionnaire was based on the questionnaire used for ChecKid, a study focusing on the behavior, health, and living circumstances of children living in the Netherlands [17,18]. The questionnaire has not been validated, but it was tested for face validity in a pilot sample of five parents with children from outside the cohort. The FPQ questionnaire included questions regarding consumption frequency of selected food items over the past two months, for seven time segments (breakfast, in the morning, lunch at school, lunch at home, in the afternoon, dinner, and in the evening). Food Frequency Questionnaires, which are more often used to assess dietary intake in cohort studies, assess habitual food intake over a specified period of time as well, but these questionnaires do not assess the timing of food intake and were, therefore, not suitable to answer the current research question. The answer possibilities for the items of the FPQ were (i) never, (ii) 0–1 times a week, (iii) 2–3 times a week, (iv) 4–5 times a week, or (v) 6–7 times a week. A preview of the questionnaire is shown in Figure S2. For analyses, these answers were recoded to continuous values (frequency per week) using the midpoint value of the categorical answer. Data were checked for face validity, including completeness, consistency, and realistic values. Based on the definition for sugar-sweetened beverages provided by the World Health Organization [19], the questions about intake of soda, fruit drinks, instant lemonade made with fruit syrup, sugar-sweetened tea, and sugar-sweetened yoghurt drinks together were considered as SSB intake. 

### 2.3. Data Analysis 

From weight and height at age 5/6 and 10/11 years, body mass index (BMI, kg/m^2^) was calculated and converted into age- and sex-specific standardized BMI-z scores with Growth Analyzer software version 3.5 (Dutch Growth Research Foundation, Rotterdam, The Netherlands). As a reference, data from the Dutch National Growth Study, 1997, were used [20]. The change in BMI-z was calculated and standardized to a period of five years by dividing the BMI-z change by the age interval between the two measurements in months, multiplied by 60. Overweight at age 5/6 and 10/11 years were defined as a BMI-z score above 1.310 for boys, and 1.244 for girls, according to Cole and Lobstein, 2012 [21]. Due to low prevalence and incidence numbers, obesity was not studied separately but was included in the category overweight. 

With the FPQ data, the total SSB consumption frequency per week was calculated. For analyses, the children were divided in quartiles based on their SSB consumption frequency. Quartile 1 (Q1) then includes the 25% of the children with the lowest consumption frequency, whereas quartile 4 (Q4) includes the 25% of the children with the highest SSB consumption frequency. Per quartile, the median consumption frequency and interquartile range (IQR, 25th–75th percentile), as well as the percentage of children consuming SSB were determined for each time segment and for the whole day. A Mantel–Haenszel test for trend was used to test whether the proportion of SSB consumers showed a significant trend over quartiles of SSB intake at different time segments. Jonckheere–Terpstra tests were performed to assess, for each time segment, whether SSB consumption frequency showed a linear trend over the quartiles. In these analyses, Kendall’s tau was determined as an estimate of the effect size of the linear trend. The larger Kendall’s tau, the larger the effect size. Additionally, depending on data characteristics Kruskal–Wallis and Fisher’s exact tests/chi-square tests were used to investigate whether other child or parental characteristics differed over the SSB quartiles. Linear regression analysis was used to investigate the association between SSB consumption frequency quartiles and change in BMI-z score (dependent variable). In the analyses, potential confounders were added to the crude model (model 1) in three steps. Model 2 was adjusted for child factors (gender, BMI-z around age five/six), model 3 was further adjusted for parental lifestyle factors (parental BMI, smoking during pregnancy, parental age), and model 4 was further adjusted for differences in socioeconomic position (parental education level).

Logistic regression analysis was used to study the association between daily SSB consumption frequency quartiles and the incidence of overweight and obesity combined (hereafter called overweight). In these models, children without overweight around age five/six were included. Models were built following the same steps as described for linear regression. For all regression analyses, quartile 1, representing the lowest SSB consumption, was used as the reference.

Variables included in the regression analyses were checked for collinearity. Paternal age was highly correlated with maternal age (r = 0.65, *p* < 0.001), and was therefore not included in the analyses. Analyses were performed in IBM SPSS Statistics 23 (SPSS, IL, USA). A two-sided significance level of *p* < 0.05 was used for all analyses.

## 3. Results

### 3.1. Baseline Characteristics

Out of 2882 ever actively participating children, 2322 children were invited to fill in the FPQ. The remaining children were not invited due to logistic issues (e.g., not screened by Community Health Services in Drenthe due to move to other province or unknown current address). Of the 1553 families that responded, 1519 filled in the FPQ completely. Of these children, 1257 met the inclusion criteria and were included in the study (Figure S1). Of all children, 50.3% were boys. Smoking during pregnancy showed a clear increase over quartiles of SSB, while parental education level showed a decrease (Table 1).

### 3.2. SSB, BMI-z, and Overweight

In a linear regression analysis adjusting for potential confounders, BMI-z change between age 5/6 and 10/11 years was between 0.066 and 0.160 SD greater in higher quartiles of daily SSB consumption frequency than in the quartile with the lowest intake (Q1) (Table 2). The prevalence of children with overweight increased from 10.5% at age 5/6 years to 17.0% at age 10/11 years. In a logistic regression analysis including children who did not have overweight at age 5/6 years (n = 1125), the adjusted odds ratio for overweight incidence was 3.12 (95% CI, 1.60–6.07) for the highest quartile (Q4) vs. the lowest quartile of SSB consumption (Q1) (Table 3).

### 3.3. SSB Consumption Patterns

**Figure 1:**
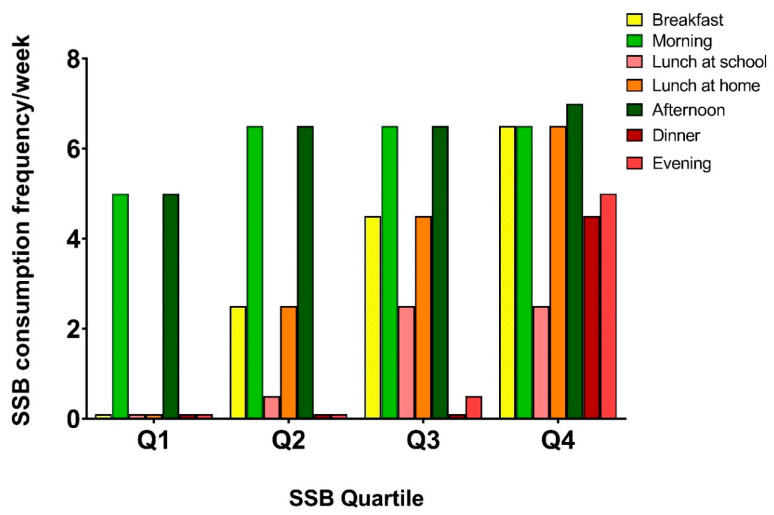
Median SSB consumption frequencies at various time segments show different trends over the quartiles of total SSB intake. The median of total SSB consumption frequency for children in Q1 is 13, in Q2 is 19, in Q3 is 25.5, and in Q4 is 34 per week. For all time segments, consumption frequency was higher in the higher quartiles (*p* < 0.001).

Differences in the timing of consumption of SSB across quartiles of total SSB intake are shown in Table 4 and Figure 1. First, Table 4 shows the percentage of children ever consuming SSBs, for each time segment of the day. For each time segment, the percentage of children consuming SSBs was significantly higher in higher quartiles of SSB consumption. Low overall consumers (Q1) and high consumers (Q4) were best distinguished by their SSB consumption behavior during main meals and in the evening, where the magnitude of the differences between quartiles was large. For example, in Q1, only 31% of children ever consume SSB during breakfast, whereas this is 98% for children in Q4. Comparable results were found for lunch at home (32% vs. 98%), dinner (17% vs. 72%), and in the evening (22% vs. 81%). The median frequency of consumption per time segment shows similar trends across quartiles (Figure 1/Table S1). With a median frequency of 5 times per week, children in Q1 consume SSBs in the morning and in the afternoon almost on a daily basis. An increase in SSB consumption frequency during breakfast and lunch characterizes children in Q2 and Q3. On top of that, children in Q4 consume SSB regularly during dinner and in the evening.

## 4. Discussion

The current study illustrated that a higher consumption frequency of SSBs is strongly related to excessive weight gain and the development of overweight, even in very young children. This higher consumption in children at elevated risk for overweight is characterized by a higher SSB consumption frequency during main meals and in the evening, whereas intake in the morning and in the afternoon is common among all children. A quarter of the children in this study only consumed SSBs in the morning and the afternoon. As most drinking occasions characterizing high SSB consumers occur in the home environment, where parental influence on dietary behaviors is profound, this uncovers an important window of opportunity. Encouraging parents to limit their children’s SSB intake during main meals and in the evening may have a high feasibility and high health potential.

In addition to previous evidence for the association of SSBs and weight gain in childhood and adulthood [4], this study showed that children in the highest quartile of SSB consumption have an approximately three times higher risk of developing overweight between age 5/6 and 10/11 years, compared to children in the lowest quartile. Previous studies within this age range were inconclusive, as some did find significant associations [11,12,13], while others did not [22,23]. As described in a review of systematic literature reviews on SSBs and obesity among children and adolescents, the discrepancy of results in this field may be related to methodological limitations, for example in study design and dietary assessment method. However, the majority of reviews did show associations between SSBs and weight gain or overweight in children and adolescents, which does support the importance of SSBs in the prevention of overweight in children [24].

In response to the increasing evidence identifying SSBs as a risk factor for excess weight gain in children, many intervention studies with diverse designs have been performed [5,6,15,24]. Unfortunately, especially in studies with a longer duration, effects on SSB intake have only been modest [5]. Therefore, the current study investigated the daily patterns of SSB intake underlying total SSB intake to help the design of interventions to limit SSB intake in children, by learning from the behaviors of children who already succeed in consuming SSBs in moderation. Our analyses regarding the timing of SSB consumption revealed that differences between children in the lowest quartile of consumption (Q1) and those in higher quartiles (Q2–Q4) were least pronounced in the morning and afternoon, when more than 90% of all children consume SSBs regularly. The differences in consumption occurred mainly during main meals and in the evening. To our knowledge, one previous study has investigated the role of the timing of consumption in SSB consumption and weight change. Our result that high SSB consumption was characterized by differences in consumption during main meals, rather than between meals, is in contrast with the results from this previous study examining daily SSB intake patterns in children [25]. In this longitudinal Canadian study following children from age 2.5 to 4.5, children who consumed SSBs between meals were more prone to overweight development. The difference in the age of the study population may offer a possible explanation for this discrepancy in results, since dietary patterns will differ between children who do and do not go to school. Nevertheless, the discrepancy may also be an indication that the results found in the current study population may be best applicable to other Western countries with similar eating habits and school systems as the Netherlands. 

From a behavioral perspective, the function of SSBs should be considered when interpreting our finding that low SSB consumers limit their consumption to the morning and to the afternoon. In the morning and in the afternoon, an SSB likely functions as a treat, while at main meals, drinks may serve to quench thirst. To quench thirst with meals, sugar-free alternatives were more often chosen by children with low overall SSB intake. During breakfast and lunch, children with lower SSB intake mainly consumed more (semi-skimmed) milk, whereas they consumed more water with their dinner (Table S2). If high consumers could follow this example, this could beneficially influence future weight change. Although substitution with water or unsweetened tea would result in the greatest reduction in caloric intake, substitution by unsweetened, low-fat milk can also benefit weight change. A previous study in children with a high predisposition for future overweight illustrated that the substitution of 100 g of sugary drinks per day with any type of milk is significantly associated with a 0.16 kg lower weight change [26]. This recommendation is also in line with most food-based dietary guidelines, such as the 2015 Dutch Dietary Guidelines [27].

Our findings that identified the main meals as occasions where low and high SSB consumers mostly differ in their behavior opens up the discussion whether it is parents or schools and day-cares that need to be addressed to limit SSB consumption. When it comes to parents, educational attainment of the parents has previously been shown to be associated with children’s dietary patterns [28]. Moreover, nutrition literacy [29], parenting style, and feeding practices play an important role [30]. Therefore, even though nutrition education can be provided to parents and children, this may not be effective enough on its own. This was illustrated in a randomized controlled trial testing a nutrition education program in elementary school children and their parents. In the intervention group, nutrition knowledge in both children and parents significantly improved after three months, while dietary patterns and BMI did not show significant improvements [31]. Other factors such as advertisement and marketing may also play a counteractive role in this situation. Products may be advertised as being for kids, or give the impression of being healthy because they contain fruit or dairy, while the items are still not the healthiest choice. It may therefore be crucial for interventions to be as explicit as possible, to ensure that capabilities at the level of the participant are not the limiting factor in the effectiveness of interventions. An example that emphasizes the potential of such interventions, comes from previous intervention studies in the home environment. In a recent Cochrane review, home-based interventions that improved access to low-calorie beverages in the home environment through the delivery of water, milk, and diet beverages were most consistently associated with a significant reduction in SSB intake, when compared to other types of intervention [32]. Additionally, providing the explicit advice to replace SSBs during main meals by these low-calorie alternatives may further aid in establishing healthier beverage consumption habits.

In addition to the role of parents and the home environment, interventions in the school or day-care environment can be of added value. Although parents may show resistance against interference in what the child is allowed to eat or drink, it may also be perceived as support for achieving the desired behavior at home. In the “Healthy Primary School of the Future”, health-promoting changes including a healthy lunch and structured physical activity session were offered and implemented using a contextual systems approach. The intervention, among others, increased water consumption, physical activity levels, and vegetable consumption and decreased BMI [33,34]. Although changes at preschool or school can help limit SSB intake or help children to get used to drinking water to quench their thirst, our study emphasizes that targeting family habits, such as SSB consumption during main meals, may prove crucial in achieving a sustainable prevention strategy for overweight.

This study has several strengths and limitations. Strengths of this study include the large study population and a long follow-up of five years. An important limitation of this study is that data on overall diet quality and energy intake were not available at this age. As SSB intake could be associated with overall diet quality, it is possible that associations of SSB and BMI or overweight were partly confounded by other aspects of the diet, such as intake of other categories of energy dense foods. Due to this residual confounding, our associations of SSB intake and anthropometric outcomes could be overestimated. Secondly, using an FPQ instead of an FFQ might lead to a different estimation of SSB intake, since this questionnaire only asked for consumption frequencies, not portions or portion sizes. Furthermore, any type of retrospective dietary intake assessment is sensitive to recall bias, which may have influenced the estimates of SSB intake in this study. Furthermore, the SSB consumption pattern may have changed between 5/6 and 10/11 years. Since a previous study found a stronger association when comparing changes in diet with changes in weight, than when comparing baseline diet with weight change, this limitation is most likely to result in an underestimation of our results [35]. The non-response to our FPQ may also have introduced bias, as it was illustrated that parental education level was lower in non-responders than in responders (Table S3). Although selection bias on socioeconomic status is also a common phenomenon in birth cohorts [36,37], the GECKO Drenthe cohort as a whole does provide a good representation of the study area (the province of Drenthe) with regard to socioeconomic status. Therefore, the overall bias in education level may still be low in comparison with other birth cohorts. However, ethnic minorities were slightly underrepresented in the cohort (9.1% in the study area’s population vs. 6.0% in the cohort), which could result in some selection bias. Lastly, our results regarding the timing of SSB consumption may not be generalizable toward populations with different school systems and eating habits, where other time points of the day may be more common for SSB consumption. 

## 5. Conclusions

To conclude, this study confirms that SSB consumption in young children is an important risk factor for prospective weight gain and overweight. More importantly, this study showed that for this Dutch population of children aged 5/6 years, the difference in SSB intake between low and high consumers is primarily established in the home environment. This emphasizes that parents play a key role in achieving a reduction in children’s SSB intake, as their influence on young children’s dietary behaviors is profound. The explicit advice to parents to substitute SSBs during main meals with drinks without added sugar, may be a way to establish a decrease in SSB intake in young children and reduce excess weight gain or overweight development.

### Implications for Future Research and Policies

The results of the current study may help to guide recommendations to limit SSB intake in young children. It was illustrated that 25% of children of the current study population naturally succeeded in limiting their consumption to two occasions per day, outside main meals. The advice to substitute SSBs with main meals by low-calorie alternatives may therefore have a high feasibility. We suggest that future studies investigate whether integrating this explicit advice into interventions in the home environment could be successful in advocating sustainable, healthier beverage consumption habits in young children.

## Figures and Tables

**Table 1:** Baseline characteristics of total study population and across quartiles of sugar-sweetened beverage (SSB) consumption frequency.

	Total (N = 1257)	Q1 (N = 313)	Q2 (N = 311)	Q3 (N = 325)	Q4 (N = 308)	*p*-Value
**Child Characteristics**						
Gender						0.499
Boy	632 (50.3)	148 (47.3)	159 (51.1)	161 (49.5)	164 (53.2)	
Girl	625 (49.7)	165 (52.7)	152 (48.9)	164 (50.5)	144 (46.8)	
Daily SSB consumption frequency	3.1 [2.3–4.2]	1.9 [1.5–2.0]	2.7 [2.5–2.9]	3.6 [3.4–3.9]	4.9 [4.6–5.5]	<0.001
Age at FPQ assessment (years)	6.0 ± 0.3	6.0 ± 0.3	6.0 ± 0.3	6.0 ± 0.3	6.1 ± 0.3	0.018
BMI-z score age 5/6 years (SD)	0.3 ± 0.8	0.3 ± 0.8	0.2 ± 0.8	0.2 ± 0.8	0.3 ± 0.9	0.412
Age at BMI measurement age 5/6 (years)	5.8 ± 0.3	5.8 ± 0.3	5.8 ± 0.3	5.8 ± 0.3	5.8 ± 0.3	0.540
BMI-z score age 10/11 years (SD)	0.2 ± 1.1	0.2 ± 1.0	0.2 ± 1.1	0.2 ± 1.0	0.4 ± 1.1	0.249
Age at BMI measurement age 10/11 (years)	10.6 ± 0.4	10.6 ± 0.4	10.6 ± 0.4	10.6 ± 0.4	10.7 ± 0.4	0.001
Overweight * prevalence at age 5/6	132 (10.5)	34 (10.9)	35 (11.3)	28 (8.6)	35 (11.4)	0.622
Overweight * prevalence at age 10/11	214 (17.0)	40 (12.8)	54 (17.4)	50 (15.4)	70 (22.7)	0.009
**Parental Characteristics**						
Age of father at birth (years)	34.4 ± 4.7	35.0 ± 4.7	34.7 ± 4.8	34.3 ± 4.8	33.8 ± 4.5	0.021
Age of mother at birth (years)	31.6 ± 4.1	32.0 ± 4.0	31.9 ± 3.9	31.6 ± 4.2	31.0 ± 4.2	0.023
BMI of father at birth (kg/m^2^)	25.4 ± 3.2	25.4 ± 3.3	25.5 ± 3.1	25.3 ± 3.2	25.7 ± 3.3	0.150
BMI of mother at birth (kg/m^2^)	24.7 ± 4.7	24.3 ± 4.5	24.7 ± 4.9	25.1 ± 4.8	24.8 ± 4.5	0.107
Paternal education level						<0.001
Low	485 (38.6)	98 (31.3)	111 (35.7)	133 (40.9)	143 (46.4)	
Middle	346 (27.5)	85 (27.2)	86 (27.7)	85 (26.2)	90 (29.2)	
High	426 (33.9)	130 (41.5)	114 (36.7)	107 (32.9)	75 (24.4)	
Maternal education level						<0.001
Low	356 (28.3)	57 (18.2)	75 (24.1)	102 (31.4)	122 (39.6)	
Middle	395 (31.4)	99 (31.6)	94 (30.2)	101 (31.1)	101 (32.8)	
High	506 (40.3)	157 (50.2)	142 (45.7)	122 (37.5)	85 (27.6)	
Maternal smoking during pregnancy	155 (12.3)	25 (8.0)	30 (9.6)	42 (12.9)	58 (18.8)	<0.001

Results presented as N (%), mean ± SD or median [25th–75th percentile], depending on data characteristics. Q1 = SSB Quartile 1 (lowest intake), Q2 = Quartile 2, Q3 = Quartile 3, Q4 = Quartile 4 (highest intake). FPQ: Food Pattern Questionnaire; BMI: body mass index; BMI-z: body mass index standardized for age and sex. * Represents overweight and obesity combined.

**Table 2:** Association of SSB consumption frequency quartile at age 5/6 years and change in BMI-z score over a 5-year period.

Model	SSB Quartile	β	95% CI	*p*-Value
**1**	Q2	0.155	0.042; 0.267	0.007
	Q3	0.098	−0.013; 0.209	0.083
	Q4	0.180	0.068; 0.293	0.002
**2**	Q2	0.149	0.037; 0.262	0.009
	Q3	0.094	−0.017; 0.205	0.097
	Q4	0.177	0.064; 0.290	0.002
**3**	Q2	0.122	0.014; 0.230	0.026
	Q3	0.065	−0.042; 0.172	0.236
	Q4	0.158	0.048; 0.267	0.005
**4**	Q2	0.122	0.014; 0.229	0.027
	Q3	0.066	−0.041; 0.174	0.227
	Q4	0.160	0.050; 0.271	0.004

β and 95% confidence intervals (CI) from linear regression analyses with SSB Quartile 1 (lowest intake) as reference, N = 1257. Model 1: Quartile of daily SSB consumption frequency; Model 2: Model 1 + Gender, BMI-z at age 5/6 y; Model 3: Model 2 + BMI father, BMI mother, smoking during pregnancy, age mother; Model 4: Model 3 + Paternal education level, maternal education level. Q1 = SSB Quartile 1 (lowest intake), Q2 = Quartile 2, Q3 = Quartile 3, Q4 = Quartile 4 (highest intake).

**Table 3:** Association of SSB consumption frequency quartile at age 5/6 years and overweight * incidence in a 5-year period. Presented numbers represented odds ratios (ORs) with 95% confidence intervals.

Model	Q1	Q2	Q3	Q4	*p*-Logistic
**1**	1 (REF)	1.95 (1.05; 3.61)	2.06 (1.13; 3.77)	2.72 (1.51; 4.92)	0.012
**2**	1 (REF)	2.57 (1.33; 4.98)	2.34 (1.23; 4.46)	3.18 (1.68; 6.00)	0.004
**3**	1 (REF)	2.36 (1.20; 4.67)	2.18 (1.12; 4.23)	3.14 (1.63; 6.04)	0.008
**4**	1 (REF)	2.30 (1.15; 4.57)	2.13 (1.09; 4.17)	3.12 (1.60; 6.07)	0.010

Results from logistic regression analyses with SSB Quartile 1 (lowest intake) as reference, N = 1125. Model 1: Quartile of daily SSB consumption frequency; Model 2: Model 1 + Gender, BMI-z at age 5/6 y; Model 3: Model 2 + BMI father, BMI mother, smoking during pregnancy, age mother; Model 4: Model 3 + Paternal education level, maternal education level. Q1 = SSB Quartile 1 (lowest intake), Q2 = Quartile 2, Q3 = Quartile 3, Q4 = Quartile 4 (highest intake). * Represents overweight and obesity combined.

**Table 4:** Percentages of children consuming SSBs at different time segments, over the 4 quartiles of SSB intake.

	Q1	Q2	Q3	Q4	*p*-Trend
Breakfast	31.3%	67.2%	86.8%	98.1%	<0.001
Morning	90.4%	97.7%	98.8%	99.4%	<0.001
Lunch at school *	46.5%	78.1%	83.8%	95.2%	<0.001
Lunch at home	31.6%	70.7%	90.5%	98.1%	<0.001
Afternoon	93.6%	96.8%	99.4%	99.7%	<0.001
Dinner	17.3%	32.2%	50.5%	72.4%	<0.001
Evening	22.4%	39.5%	56.0%	81.2%	<0.001
Total	99.0%	100.0%	100.0%	100.0%	0.030

* Of 764 children who have lunch at school at least once a week, median + interquartile range for frequency of lunch at school = 2 [1; 3]. N = 1257. Q1 = SSB Quartile 1 (lowest intake), Q2 = Quartile 2, Q3 = Quartile 3, Q4 = Quartile 4 (highest intake).

## Data Availability

The datasets used and/or analyzed during the current study are available from the corresponding author on reasonable request.

## Supplementary Materials

The following are available online at https://www.mdpi.com/2072-6643/12/8/2486/s1, Figure S1: Flow-chart of exclusion, Figure S2: Preview of food pattern questionnaire, Table S1: Median SSB consumption frequencies per SSB quartile at different time segments, Table S2: Median consumption frequencies of non-sugar sweetened beverages per SSB quartile at different time segments, Table S3: Characteristics of FPQ responders and non-responders.
