# Supplementary material for: Young Children’s Sugar-Sweetened Beverage Consumption and 5-Year Change in BMI: Lessons Learned from the Timing of Consumption
Source: Nutrients. 2020 Aug 18;12(8):2486. doi: 10.3390/nu12082486 (PMC7468701; doi:10.3390/nu12082486)
Supplement: Supplementary file 1 [file nutrients-12-02486-s001.pdf]

# Additional materials

**Figure S1:** Flow-chart of excluded participants. (ICF = Informed Consent Form, FPQ = food pattern questionnaire).

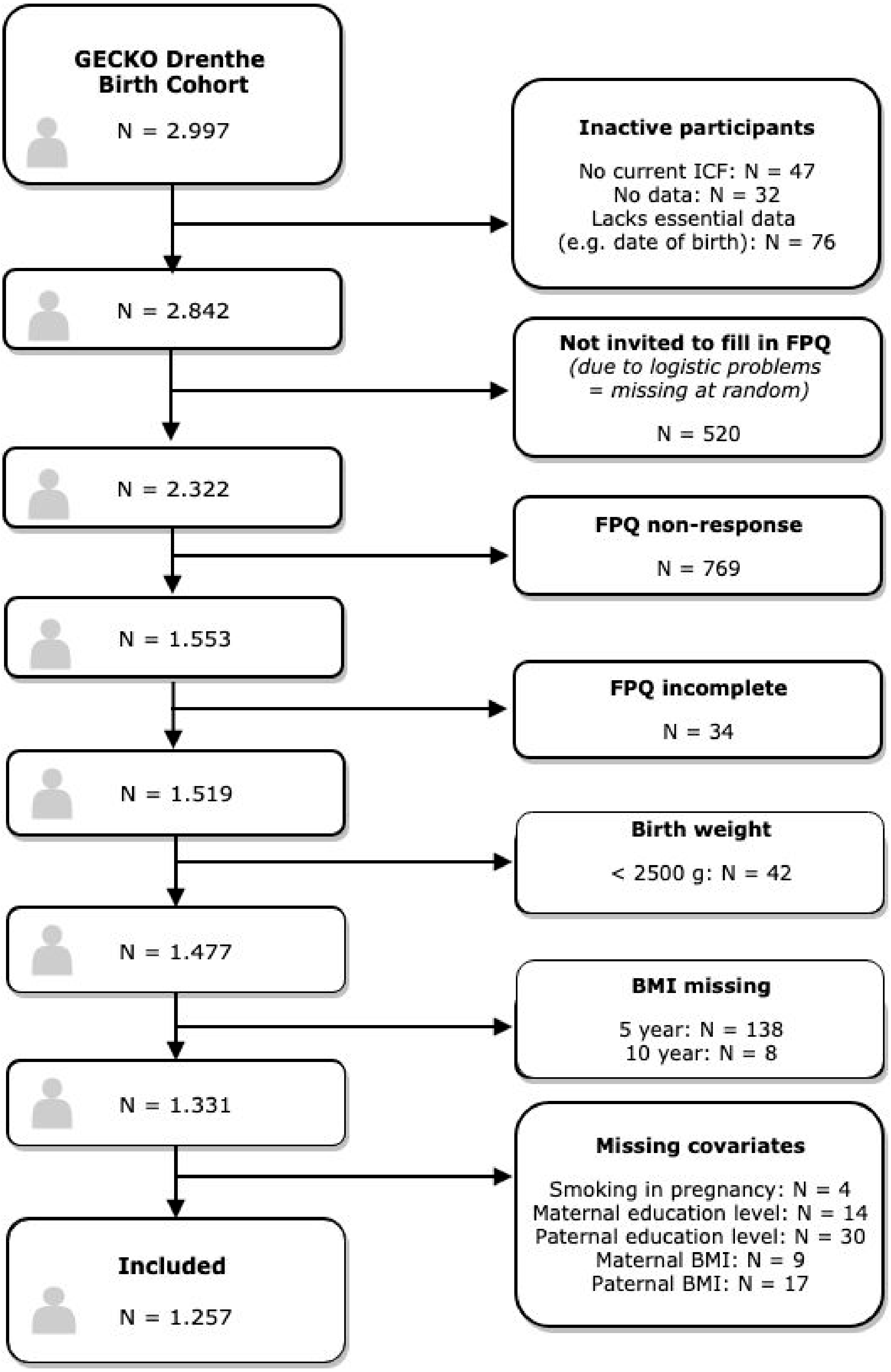

**Figure S2: Preview of food pattern questionnaire.** The same question about beverages was asked in the morning, during lunch at school, lunch at home, in the afternoon, dinner, and in the evening.

What does your child usually drink during breakfast, and how often? **(tick one box in each line)**

| | Never | 0-1 times a week | 2-3 times a week | 4-5 times a week | 6-7 times a week |
|---|---|---|---|---|---|
| Nothing | O | O | O | O | O |
| Water | O | O | O | O | O |
| Milk or buttermilk | O | O | O | O | O |
| Yoghurtdrink, sweetened dairy drink, chocolate milk | O | O | O | O | O |
| Soda, regular | O | O | O | O | O |
| Soda, light | O | O | O | O | O |
| Fruit juice, store bought | O | O | O | O | O |
| Fruit juice, homemade | O | O | O | O | O |
| Instant lemonade made with fruit syrup | O | O | O | O | O |
| Tea without sugar | O | O | O | O | O |
| Tea with sugar | O | O | O | O | O |
| Tea with milk and sugar | O | O | O | O | O |

**Table S1:** Median [IQR] weekly consumption frequency of sugar-sweetened beverages at different time segments, over the 4 quartiles of SSB intake.

| | **Q1** | **Q2** | **Q3** | **Q4** | **P-trend** | **Kendall's tau** |
|---|---|---|---|---|---|---|
| Breakfast | 0<br>[0 - 0,5] | 2,5<br>[0 - 4,5] | 4,5<br>[2,5 - 6,5] | 6,5<br>[5 - 7] | **<0.001** | 0.59 |
| Morning | 5<br>[4,5 - 6,5] | 6,5<br>[4,5 - 6,5] | 6,5<br>[4,5 – 6,5] | 6,5<br>[5 - 7] | **<0.001** | 0.27 |
| Lunch at school* | 0<br>[0 - 0,5] | 0,5<br>[0,5 - 2,5] | 2,5<br>[0,5 - 2,5] | 2,5<br>[1 - 3] | **<0.001** | 0.38 |
| Lunch at home | 0<br>[0 - 0,5] | 2,5<br>[0 - 4,5] | 4,5<br>[2,5 - 5,5] | 6,5<br>[4,5 - 7] | **<0.001** | 0.60 |
| Afternoon | 5<br>[4,5 - 6,5] | 6,5<br>[4,5 - 6,5] | 6,5<br>[5 - 7] | 7<br>[6,5 - 9] | **<0.001** | 0.33 |
| Dinner | 0<br>[0 - 0] | 0<br>[0 - 1] | 0<br>[0 - 4,5] | 4,5<br>[0 - 6,5] | **<0.001** | 0.40 |
| Evening | 0<br>[0 - 0] | 0<br>[0 - 2,5] | 0,5<br>[0 - 4,5] | 5<br>[2,5 - 6,5] | **<0.001** | 0.44 |
| Total | 13<br>[10,5 - 14] | 19<br>[17,5 - 20,5] | 25,5<br>[23,5 - 27,5] | 34<br>[32 – 38,5] | **<0.001** | 0.87 |

** of 764 children who have lunch at school at least once a week, median + IQR frequency of lunch at school = 2 days [1 ; 3]*

N=1257

**Table S2:** Median [IQR] weekly consumption frequency of non-sugar-sweetened beverages at different time segments, over the 4 quartiles of SSB intake.

| | Q1 | Q2 | Q3 | Q4 | P-trend | Kendall's tau |
|---|---|---|---|---|---|---|
| **Breakfast** | | | | | | |
| Nothing | 0 [0 - 0] | 0 [0 - 0] | 0 [0 - 0] | 0 [0 - 0] | 0.445 | -0.020 |
| Water | 0 [0 - 0] | 0 [0 - 0] | 0 [0 - 0] | 0 [0 - 0] | 0.002 | -0.079 |
| Milk* | 6,5 [2,5 - 6,5] | 4,5 [0 - 6,5] | 0 [0 - 4,5] | 0 [0 - 0] | <0.001 | -0.428 |
| Tea without sugar | 0 [0 - 0,5] | 0 [0 - 0,5] | 0 [0 - 0] | 0 [0 - 0] | 0.001 | -0.082 |
| **In the morning** | | | | | | |
| Nothing | 0 [0 - 0] | 0 [0 - 0] | 0 [0 - 0] | 0 [0 - 0] | 0.902 | 0.003 |
| Water | 0 [0 - 0] | 0 [0 - 0] | 0 [0 - 0] | 0 [0 - 0] | 0.031 | -0.054 |
| Milk* | 0 [0 - 0] | 0 [0 - 0] | 0 [0 - 0] | 0 [0 - 0] | <0.001 | -0.131 |
| Tea without sugar | 0 [0 - 0] | 0 [0 - 0] | 0 [0 - 0] | 0 [0 - 0] | 0.333 | 0.025 |
| **Lunch at school**** | | | | | | |
| Nothing | 0 [0 - 0] | 0 [0 - 0] | 0 [0 - 0] | 0 [0 - 0] | 0.712 | -0.012 |
| Water | 0 [0 - 0] | 0 [0 - 0] | 0 [0 - 0] | 0 [0 - 0] | 0.766 | 0.010 |
| Milk* | 0,5 [0 - 2,5] | 0 [0 - 2,5] | 0 [0 - 0,5] | 0 [0 - 0,4] | <0.001 | -0.267 |
| Tea without sugar | 0 [0 - 0] | 0 [0 - 0] | 0 [0 - 0] | 0 [0 - 0] | 0.060 | 0.062 |
| **Lunch at home** | | | | | | |
| Nothing | 0 [0 - 0] | 0 [0 - 0] | 0 [0 - 0] | 0 [0 - 0] | 0.053 | -0.050 |
| Water | 0 [0 - 0] | 0 [0 - 0] | 0 [0 - 0] | 0 [0 - 0] | 0.322 | -0.025 |
| Milk* | 4,5 [2,5 - 6,5] | 2,5 [0 - 4,5] | 0 [0 - 2,5] | 0 [0 - 0,5] | <0.001 | -0.411 |
| Tea without sugar | 0 [0 - 0] | 0 [0 - 0] | 0 [0 - 0] | 0 [0 - 0] | 0.040 | 0.052 |
| **In the afternoon** | | | | | | |
| Nothing | 0 [0 - 0] | 0 [0 - 0] | 0 [0 - 0] | 0 [0 - 0] | 0.146 | -0.037 |
| Water | 0 [0 - 2,5] | 0 [0 - 0,5] | 0 [0 - 0] | 0 [0 - 0] | <0.001 | -0.114 |
| Milk* | 0 [0 - 0] | 0 [0 - 0] | 0 [0 - 0] | 0 [0 - 0] | 0.017 | -0.061 |
| Tea without sugar | 0 [0 - 0,5] | 0 [0 - 0] | 0 [0 - 0] | 0 [0 - 0] | <0.001 | -0.088 |
| **Dinner** | | | | | | |
| Nothing | 0 [0 - 4,5] | 0 [0 - 4,5] | 0 [0 - 4,5] | 0 [0 - 0,5] | 0.005 | -0.069 |
| Water | 2,5 [0 - 4,5] | 0 [0 - 2,5] | 0 [0 - 2,5] | 0 [0 - 2,5] | <0.001 | -0.188 |
| Milk* | 0 [0 - 0] | 0 [0 - 0] | 0 [0 - 0] | 0 [0 - 0] | <0.001 | -0.097 |
| Tea without sugar | 0 [0 - 0] | 0 [0 - 0] | 0 [0 - 0] | 0 [0 - 0] | 0.069 | 0.047 |
| **In the evening** | | | | | | |
| Nothing | 0 [0 - 6,5] | 0 [0 - 4,5] | 0 [0 - 4,5] | 0 [0 - 0] | <0.001 | -0.191 |
| Water | 0,5 [0 - 2,5] | 0 [0 - 2,5] | 0 [0 - 2,5] | 0 [0 - 0,5] | <0.001 | -0.125 |
| Milk* | 0 [0 - 0] | 0 [0 - 0] | 0 [0 - 0] | 0 [0 - 0] | <0.001 | -0.123 |
| Tea without sugar | 0 [0 - 0] | 0 [0 - 0] | 0 [0 - 0] | 0 [0 - 0] | 0.319 | 0.025 |

** Mostly semi-skimmed milk, 1.5-1.8% fat*

*** of 764 children who have lunch at school at least once a week, median + IQR frequency of lunch at school = 2 days [1 ; 3]. N=1257*

**Table S3:** Baseline characteristics of families that were not invited to fill out the FPQ due to logistic problems, families who did not respond to the invitation and families who did respond.

| | **Not invited** (N=520) | **Non-response** (N=769) | **Response** (N=1.553) | **P-value** |
|---|---|---|---|---|
| **Child characteristics** | | | | |
| Gender | | | | 0.981 |
| *Boy* | 262 (50.5) | 385 (50.1) | 784 (50.5) | |
| *Girl* | 257 (49.5) | 384 (49.9) | 769 (49.5) | |
| Daily SSB consumption frequency | N/A | N/A | 3.1 [2.2 - 4.2] | N/A |
| Age at FPQ assessment (y) | N/A | N/A | 6 ± 0.4 | N/A |
| BMI-z score age 5/6 (y) | N/A | 0.3 ± 0.9 | 0.3 ± 0.8 | 0.411 |
| Age at BMI 5/6 (SD) | N/A | 5.9 ± 0.4 | 5.8 ± 0.3 | <0.001 |
| BMI-z score age 10/11 (SD) | 0.2 ± 1.1 | 0.4 ± 1 | 0.2 ± 1.1 | 0.003 |
| Age at BMI 10/11 (SD) | 10.9 ± 0.6 | 10.7 ± 0.5 | 10.6 ± 0.4 | <0.001 |
| Overweight prevalence age 5/6 (y) | N/A | 74 (10.1) | 161 (10.5) | 0.825 |
| Overweight prevalence age 10/11 (y) | 25 (15.3) | 128 (20.4) | 230 (16.3) | 0.066 |
| **Parental characteristics** | | | | |
| Age father at birth (y) | 33.7 ± 5.2 | 33.8 ± 5.2 | 34.4 ± 4.8 | 0.002 |
| Age mother at birth (y) | 30.7 ± 4.7 | 30.8 ± 4.7 | 31.6 ± 4.2 | <0.001 |
| BMI father at birth (kg/m$^2$) | 25.5 ± 3.4 | 25.9 ± 3.5 | 25.4 ± 3.2 | 0.102 |
| BMI mother at birth (kg/m$^2$) | 24.6 ± 4.3 | 24.8 ± 5.0 | 24.7 ± 4.7 | 0.983 |
| Paternal education level | | | | <0.001 |
| *Low* | 218 (50.2) | 319 (51.5) | 596 (40.0) | |
| *Middle* | 86 (19.8) | 142 (22.9) | 394 (26.4) | |
| *High* | 130 (30) | 158 (25.5) | 500 (33.6) | |
| Maternal education level | | | | <0.001 |
| *Low* | 185 (41) | 312 (47.3) | 468 (30.7) | |
| *Middle* | 102 (22.6) | 159 (24.1) | 467 (30.6) | |
| *High* | 164 (36.4) | 189 (28.6) | 589 (38.6) | |
| Maternal smoking during pregnancy | 82 (16.4) | 155 (20.4) | 200 (12.9) | <0.001 |

*Results presented as N(%), mean ± SD or median [25$^{th}$ – 75t percentile], depending on data characteristics.*
